# Plasma Inter-Alpha-Trypsin Inhibitor Heavy Chains H3 and H4 Serve as Novel Diagnostic Biomarkers in Human Colorectal Cancer

**DOI:** 10.1155/2019/5069614

**Published:** 2019-07-31

**Authors:** Xiao Jiang, Xiao-Yan Bai, Bowen Li, Yanan Li, Kangkai Xia, Miao Wang, Shujing Li, Huijian Wu

**Affiliations:** ^1^Key Laboratory of Protein Modification and Disease of Liaoning Province, School of Life Science and Biotechnology, Dalian University of Technology, No. 2 Ling Gong Road, Dalian 116024, China; ^2^Department of Gastroenterology and Hepatology, Dalian Municipal Central Hospital Affiliated to Dalian Medical University, No. 826 Xi Nan Road, Dalian 116033, China

## Abstract

**Objective:**

Inter-alpha-trypsin inhibitor heavy chain H3 (ITIH_3_) and inter-alpha-trypsin inhibitor heavy chain H4 (ITIH_4_) are heavy chains of protein members belonging to the ITI family, which was associated with inflammation and carcinogenesis. However, the diagnostic value of ITIH_3_ and ITIH_4_ in human colorectal cancer (CRC) remains unknown.

**Methods:**

In total, 101 CRC patients and 156 healthy controls were enrolled. The concentrations of ITIH_3_ and ITIH_4_ proteins in plasma samples of participants were assessed using enzyme-linked immunosorbent assay. ITIH_3_ and ITIH_4_ expressions in human CRC tissues were additionally assessed via immunohistochemical staining (IHC). Receiver operating characteristic (ROC) was applied to estimate the diagnostic power of the two proteins, and the net reclassification improvement (NRI) was adopted to evaluate the incremental predictive ability of ITIH_3_/ITIH_4_ when added to the tissue inhibitor of metalloproteinase-1 (TIMP-1).

**Results:**

The plasma concentration of ITIH_3_ in CRC patients (median: 4.370 *μ*g/mL; range: 2.152–8.170 *μ*g/mL) was significantly lower than that in healthy subjects (median: 4.715 *μ*g/mL; range: 2.665–10.257 *μ*g/mL; *p* < 0.001), while the ITIH_4_ plasma level in subjects with CRC (median: 0.211 *μ*g/mL; range: 0.099–0.592 *μ*g/mL) was markedly increased relative to that in the control group (median: 0.134 *μ*g/mL; range: 0.094–0.460 *μ*g/mL, *p* < 0.001). Consistently, IHC score assessment showed a dramatic reduction in ITIH_3_ expression and, conversely, upregulation of ITIH_4_ in colorectal carcinoma specimens relative to adjacent normal colorectal tissues (*p* < 0.001 in both cases). The area under the curve (AUC) of the ROC for ITIH_4_ (AUC = 0.801, 95% CI: 0.745–0.857) was higher than that for ITIH_3_ (AUC = 0.638, 95% CI: 0.571–0.704, both *p* values < 0.001). The AUC of the ROC for combined ITIH_3_ and ITIH_4_ was even higher than that for carcinoembryonic antigen. NRI results showed that combining ITIH_3_ and ITIH_4_ with TIMP-1 significantly improved diagnostic accuracy (NRI = 17.12%, *p* = 0.002) for CRC patients compared to TIMP-1 alone.

**Conclusions:**

Circulating ITIH_3_ and ITIH_4_ levels are associated with carcinogenesis in CRC, supporting their potential diagnostic utility as surrogate biomarkers for colorectal cancer detection.

## 1. Introduction

Colorectal cancer (CRC) is the third most commonly diagnosed cancer type in both men and women and the fourth leading cause of cancer-related mortality worldwide [1–3], causing more than 50,000 deaths in the USA each year [4]. The recent years have seen a continuing increase in the incidence and mortality of CRC in China [5, 6].

CRC often appears to develop and progress slowly over the years. In many cases, there is an initial noninvasive polyp stage in the setting of chronic inflammation, which presents a more convenient step for prevention screening relative to many other solid malignancies. Current screening strategies, such as the fecal occult blood test (FOBT), fecal immunochemical testing, and colonoscopy, have improved the effectiveness of CRC detection [7]. However, on average, only 65% of the elderly population have undergone CRC screening tests in the United States [8]. An effective alternative strategy may be to develop more specific biomarkers detectable in the peripheral blood for accurate and reliable detection of CRC.

The inter-alpha-trypsin inhibitor (ITI) family proteins which were originally isolated from human plasma are plasma serine protease inhibitor proteins [9]. ITIs are composed of one light chain (bikunin) and five homologous heavy chains [10]. The inter-alpha-trypsin inhibitor heavy chains (ITIHs) are involved in inflammation as well as tumorigenic and metastatic processes. The proteins are covalently linked to hyaluronic acid (HA), a major component of the extracellular matrix. Since HA linking and extracellular matrix stability are strongly dependent on ITIHs, dysregulation of *ITIH* family members could influence the vascularization process during tumor development [11]. In two proteomic studies, ITIH_3_/ITIH_4_, as one of the serum differential proteins, was detected from the patients of hepatocellular cancer or gastric cancer [12, 13], indicating a potential relation of ITIH_3_/ITIH_4_ to digestive system cancers. It has been reported that ITIH_3_ and ITIH_4_ serve as candidate plasma proteins indicative of early-stage intestinal cancer in a mouse model [14]. Additionally, ITIH_4_ was shown to be upregulated in plasma of mice with severe colitis [15] and had high sensitivity and specificity in the identification of early-stage colon adenoma [16]. Furthermore, the ITIH_4_ level was significantly elevated in THE serum of patients growing early colorectal adenomas, which was identified as a premalignant state [17]. Considering that there have been no studies aiming at ITIH_3_/ITIH_4_ in the human plasma of CRC, we therefore selected the two proteins as plasma biomarkers to determine their association with CRC risk.

In this case control study, we examined the levels of ITIH_3_ and ITIH_4_ proteins in human plasma and evaluated their diagnostic value as biomarkers for CRC. Expressions of ITIH_3_ and ITIH_4_ proteins in colorectal cancer and adjacent normal tissues were additionally examined via immunohistochemical (IHC) analysis.

## 2. Materials and Methods

### 2.1. Participants

In total, 101 patients diagnosed with CRC and treated between January 2017 and July 2018 at the Dalian Municipal Central Hospital Affiliated to Dalian Medical University (Dalian, China) participated in the study. CRC was diagnosed based on pathological findings from tissue specimens of patients. Staging information was determined histopathologically and combined with various imaging modalities, such as computed tomography, magnetic resonance imaging, and clinical information. According to disease stages based on the American Joint Committee on Cancer staging system [18], colorectal cancer patients were divided into two subgroups: nonmetastatic (stages I to III) and metastatic (stage IV).

The 156 healthy controls were randomly selected from participants subjected to health screening during the same period at the Medical Examination Center of Dalian Municipal Central Hospital. Our study was conducted with the human subjects' understanding and consent and was approved by the ethics committee of Dalian Municipal Central Hospital (no. YN2017-034-01). All work was carried out in accordance with the Helsinki declaration.

### 2.2. Colorectal Cancer Plasma and Tissue Samples

All 257 blood samples used in the study were collected into EDTA-containing tubes, which were centrifuged at 3000 × g for 15 min to separate blood cells. Plasma was collected into another tube and stored at −80°C until experimental use.

For immunohistochemical analysis, matched malignant and adjacent normal colorectal tissues were obtained from patients who accepted surgery. Tissue specimens were fixed in 10% buffered formalin solution, dehydrated, and embedded in paraffin.

### 2.3. Enzyme-Linked Immunosorbent Assay (ELISA) for ITIH_3_, ITIH_4_, and Tissue Inhibitor of Metalloproteinase-1 (TIMP-1) Plasma Levels

The plasma levels of ITIH_3_, ITIH_4_, and TIMP-1 were detected independently by two researchers. Commercial ELISA kits (Lifespan Biosciences Inc., Seattle, WA, USA) were employed for identifying the plasma concentrations of ITIH_3_ (catalog no. LS-F7346), ITIH_4_ (catalog no. LS-F6535), and TIMP-1 (catalog no. LS-F24684). All steps were conducted according to the manufacturer's instructions: (1) Standard, blank, and experimental samples (pretreated with sample diluents) were added to individual wells of 96-well microplates precoated with the target-specific capture antibodies (anti-ITIH_3_ or anti-ITIH_4_) and incubated for 1 h at 37°C. (2) After aspirating the liquid of each well, a biotin-conjugated detection secondary antibody was added to the wells and incubated for 1 h at 37°C. (3) The liquid was aspirated again and washed three times per well with Wash Buffer solution. Next, the fluid in each well was removed, and after washing three times with Wash Buffer, each well was filled with HRP conjugate for 30 min at 37°C. (4) Similarly, after washing at least five times, wells were incubated with tetramethyl benzidine (TMB) substrate for 10–20 min at 37°C. (5) Finally, 50 *μ*L stop solution was used to terminate the color reaction, and the optical density value of each well was determined immediately using a microplate reader at a wavelength of 450 nm. The concentrations of ITIH_3_ and ITIH_4_ (*μ*g/mL) in each well were calculated using the standard curve.

### 2.4. Determination of Carcinoembryonic Antigen (CEA) Plasma Concentrations

CEA is the most widely used biomarker for CRC in the clinical setting. The CEA plasma level was analyzed using the specific electrochemiluminescence immunoassay and measured by the Roche Cobas e601 system (Roche Diagnostics Inc., Indianapolis, IN, USA).

### 2.5. IHC Staining

Paraffin block-embedded human tissues were cut into 5 *μ*m sections using a microtome. IHC was conducted with primary ITIH_3_ (Proteintech Group Inc., Chicago, IL, USA, catalog no. 21247-1-AP) and ITIH_4_ (Proteintech Group Inc., Chicago, IL, USA, catalog no. 24069-1-AP) antibodies at a 1 : 500 dilution. The antigen-antibody complex was visualized using diaminobenzidine chromogen. The immunoreactivity intensity of ITIH_3_ or ITIH_4_ in cancer and adjacent normal colorectal tissues was evaluated via light microscopy. Immunohistochemical staining intensity of ITIH_3_ or ITIH_4_ was scored as negative (0), weak (1), moderate (2), and strong (3), and the percentage of positive cells as 5% (0), 5–30% (1), 31–50% (2), and >50% (3); the IHC score of each slide was calculated by multiplying these two values (ranging from 0 to 9). This is the method of Zhao et al., and the method description partly reproduces their wording [19]. All individuals who donated tissues for this study provided written informed consent. A total of 20 colorectal carcinoma and adjacent normal colorectal tissue specimens were analyzed.

### 2.6. Statistical Analysis

Statistical analysis was performed with SPSS software (SPSS Inc., SPSS Standard version 22.0, Chicago, IL, USA) and GraphPad Prism (GraphPad Software Inc., GraphPad Prism version 5.01, La Jolla, CA, USA). Data which did not follow normal distribution based on the Kolmogorov-Smirnov test are presented as median values with ranges. Nonparametric statistical analyses (the Mann–Whitney *U* or the Kruskal-Wallis test) were used to compare the differences between two independent groups. The ages of individuals in the two groups, which followed a normal distribution, were presented as the mean and standard deviation (SD) and compared using Student's *t*-test. Pearson's chi-squared test was adopted to evaluate the differences in characteristics of patients compared with healthy controls. Receiver operating characteristic (ROC) curves were generated to estimate the sensitivity and specificity of the biomarkers in diagnosing CRC. A binomial logistic regression model was fitted to combine the diagnostic performance of different biomarkers. The net reclassification improvement (NRI) was applied to estimate the incremental predictive ability of ITIH_3_/ITIH_4_ based when added to TIMP-1. The value of NRI is the overall reclassification sum of differences, in proportions of individuals reclassified upward minus the proportion reclassified downward for people who developed events and the proportion of individuals moving downward minus the proportion moving upward for those who did not develop events, and the statistical significance of the overall improvement is assessed with an asymptotic test, as described by Pencina et al. [20]. The survival rates were calculated by the Kaplan-Meier method, and differences between survival curves were analyzed by the log-rank tests. IHC scores in different groups were compared using a paired *t*-test. A two-sided probability value of less than 0.05 was considered statistically significant.

## 3. Results

### 3.1. Baseline Characteristics of Patients and Controls

The clinicopathologic characteristics of patients and control subjects are described in Table 1. A total of 101 patients (57 male and 44 female) with CRC were diagnosed between January 2017 and July 2018 at the Dalian Municipal Central Hospital Affiliated to Dalian Medical University. The mean patient age was 61.089 ± 8.505 years. One hundred and fifty-six noncancer subjects of similar ages with the same ethnicity were selected from the checkup population of the hospital as the normal control group. The mean age of the control group was 59.359 ± 7.792 years. No significant differences between the patient and control groups were identified in terms of sex (*p* = 0.363), age (*p* = 0.095), smoking (*p* = 0.172), or drinking (*p* = 0.345) status.

### 3.2. Relationship between Plasma ITIH_3_ and ITIH_4_ Expression Patterns of CRC Patients and Clinicopathological Features of Tumors

The concentrations of ITIH_3_ and ITIH_4_ were estimated in all preoperative plasma samples with the aid of ELISA. Moreover, to determine the effects of clinicopathological features on the concentration of ITIH_3_ or ITIH_4_ in the case group, the associations between ITIH_3_ and ITIH_4_ concentrations and clinicopathological features were analyzed in CRC patients. No statistical significances were detected in the mean plasma levels of ITIH_3_ or ITIH_4_ between various subgroups stratified by clinical characteristics, and all corresponding *p* values were greater than 0.05 (Table 2). Our results suggest that clinical features have no obvious influence on the plasma ITIH_3_ or ITIH_4_ concentrations in CRC patients.

### 3.3. Significant Alterations in Plasma ITIH_3_ and ITIH_4_ Expressions in CRC Patients

**Figure 1:**
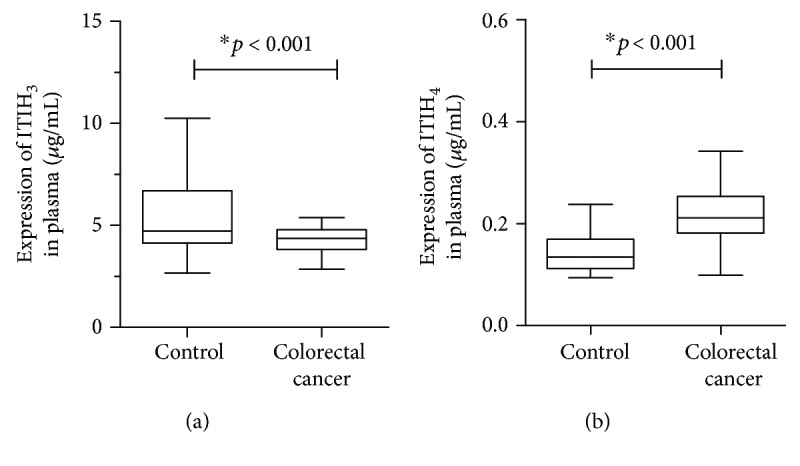
The plasma expression of inter-alpha-trypsin inhibitor heavy chain H3/H4 (ITIH_3_/ITIH_4_) in colorectal cancer (CRC) patients (*n* = 101) was compared with that of healthy subjects (*n* = 156). (a) The box plot showed the distributions of the plasma ITIH_3_ level in CRC patients and the normal controls. (b) The other box plot described the ITIH_4_ expression in the plasma of CRC patients relative to the normal subjects.

Next, we evaluated the expression levels of ITIH_3_ and ITIH_4_ in the plasma of both CRC patients and normal control subjects to establish their utility as potential biomarkers for CRC detection. The statistical results for comparison of the ITIH_3_ or ITIH_4_ plasma concentrations between CRC patients and controls are shown in Table 3. The plasma ITIH_3_ level in CRC patients (median: 4.370 *μ*g/mL; range: 2.152–8.170 *μ*g/mL) was significantly lower than that in the control group (median: 4.715 *μ*g/mL; range: 2.665–10.257 *μ*g/mL; *p* < 0.001; Figure 1(a)). The median plasma levels of ITIH_4_ in colorectal cancer patients and healthy controls were 0.211 *μ*g/mL (range: 0.099–0.592 *μ*g/mL) and 0.134 *μ*g/mL (range: 0.094–0.460 *μ*g/mL), respectively. A box plot (Figure 1(b)) further revealed that ITIH_4_ expression in the plasma of CRC patients is significantly upregulated, compared with that in normal subjects (*p* < 0.001). Our data collectively suggest that plasma ITIH_3_ and ITIH_4_ may be useful as biomarkers for differentiating CRC patients from healthy subjects.

### 3.4. Diagnostic Efficiency of ITIH_3_/ITIH_4_ for CRC Patients

We conducted ROC curve analysis to determine the sensitivity and specificity of ITIH_3_ and ITIH_4_ in the detection of CRC. The ROC curve of CEA was additionally obtained to compare the efficacy of these two plasma proteins with that of the classical clinical biomarker in CRC diagnosis. Recently, emerging evidence has shown that TIMP-1 is a promising biomarker in the early diagnosis of CRC and more superior to CEA [21]. Therefore, we also conducted the ROC curve for TIMP-1 and consider it as an important comparison.

**Figure 2:**
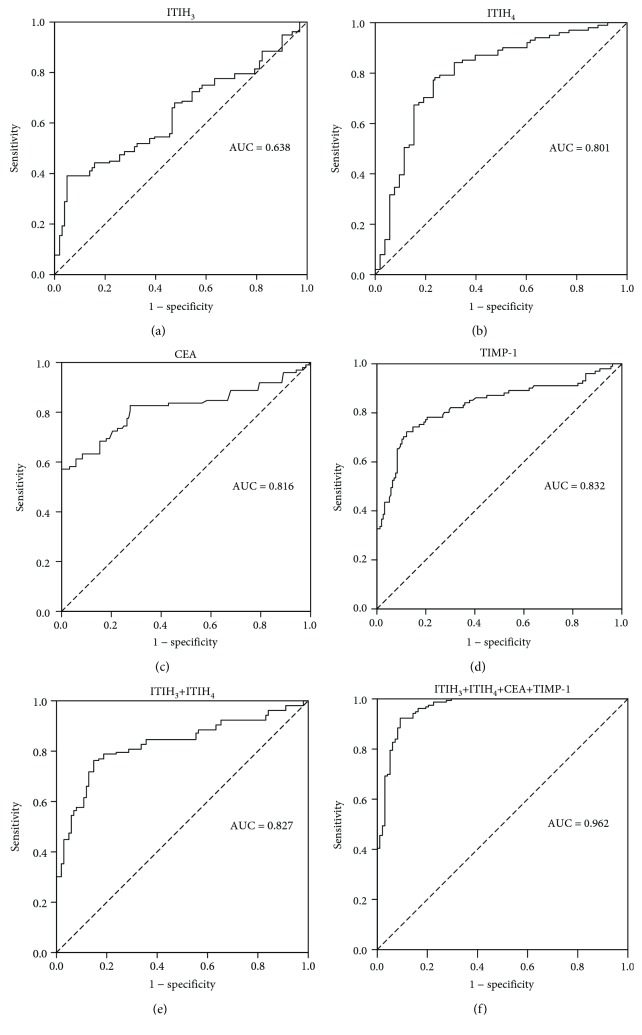
The receiver operating characteristic (ROC) curves were plotted for the biomarkers. (a) The ROC curve for ITIH_3_ (area under the curve (AUC) = 0.638). (b) The ROC curve for ITIH_4_ (AUC = 0.801). (c) The ROC curve for CEA (AUC = 0.816). (d) The ROC curve for TIMP-1 (AUC = 0.832). (e) The diagnostic accuracy of ITIH_3_ and ITIH_4_ combinations was assessed by a logistic regression model. The ROC curve for combined ITIH_3_ and ITIH_4_ (AUC = 0.827). (f) The combinations of CEA, TIMP-1, ITIH_3_, and ITIH_4_ yielded the highest diagnostic accuracy (AUC = 0.962).

The area under the curve (AUC) for ITIH_4_ (AUC = 0.801, 95% confidence interval (CI): 0.745–0.857, *p* < 0.001) (Figure 2(b)) was higher than that for ITIH_3_ (AUC = 0.638, 95% CI: 0.571–0.704, *p* < 0.001) (Figure 2(a)) while those for CEA and TIMP-1 were 0.816 (95% CI: 0.754–0.878, *p* < 0.001) (Figure 2(c)) and 0.832 (95% CI: 0.776–0.888, *p* < 0.001) (Figure 2(d)).

Using a logistic regression model, the diagnostic capabilities of ITIH_3_ and ITIH_4_ were combined, generating a ROC curve, with an AUC of 0.827 (95% CI: 0.776–0.877, *p* < 0.001) (Figure 2(e)), which was even higher than that of CEA, indicating that the combined two biomarkers could be representative of greater effectiveness in disease diagnosis. In particular, the combined ROC analysis of TIMP-1, CEA, ITIH_3_, and ITIH_4_ revealed the highest diagnostic accuracy (AUC = 0.962, 95% CI: 0.940–0.985, *p* < 0.001) (Figure 2(f)) was at the cutoff value of 0.705, with the corresponding sensitivity of 0.917 and specificity of 0.908.

ROC analyses indicated that plasma ITIH_3_ and ITIH_4_ levels may be successfully employed to discriminate patients with CRC from control subjects and ITIH_3_/ITIH_4_ has the potential to serve as a diagnostic marker in colorectal cancer. All the results of ROC analysis are shown in Table 4.

### 3.5. NRI Analysis for Combining ITIH_3_/ITIH_4_ and TIMP-1

Since the ROC curve of TIMP-1 showed the highest diagnostic accuracy among the overall biomarkers we detected, we chose TIMP-1 as a reliable biomarker for further analysis. The NRI analysis was conducted to estimate the incremental predictive ability combining ITIH_3_/ITIH_4_ with TIMP-1 compared to TIMP-1 alone.

The NRI for reclassification showed significant improvements for CRC detection when ITIH_3_ was added to TIMP-1 (NRI = 13.6%, *p* = 0.006). Additionally, there was a relatively small improvement in the predictive value of ITIH_4_ combined with TIMP-1 compared to TIMP-1 alone (NRI = 6.2%, *p* = 0.241). Finally, we combined both ITIH_3_ and ITIH_4_ into TIMP-1 and yielded the highest NRI of 17.1% (*p* = 0.002).

Taken together, these results suggested that in CRC patients, ITIH_3_/ITIH_4_ could significantly add the diagnostic accuracy beyond that provided by TIMP-1 alone.

### 3.6. Altered Expression of ITIH_3_/ITIH_4_ in Human CRC Tissues

**Figure 3:**
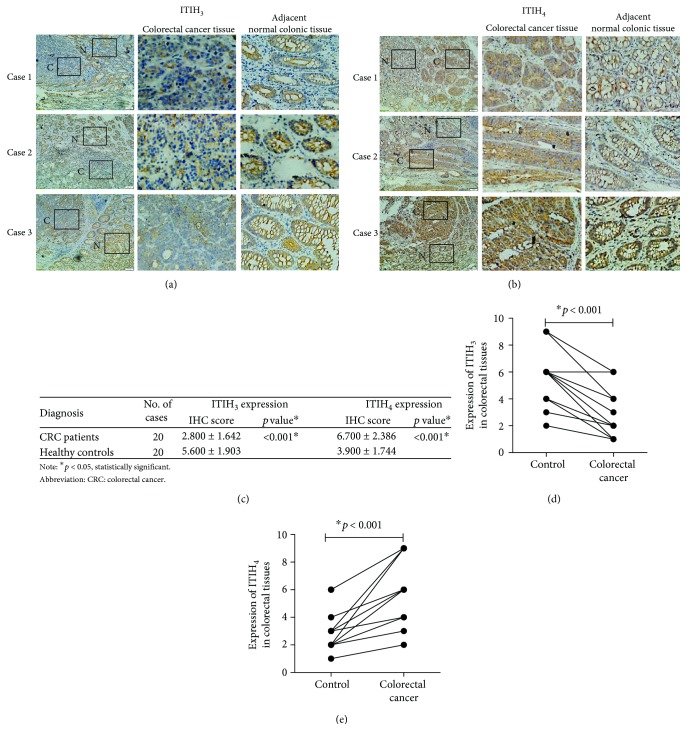
The expressions of ITIH_3_ or ITIH_4_ in human CRC tissues and their adjacent normal colorectal tissues were analyzed by immunohistochemical (IHC) staining. (a) The expressions of ITIH_3_ in CRC tissues and the normal colorectal tissues. The boxed areas lined with black color in the left images of (a, b) were magnified in the middle and right ones. N: adjacent normal tissue (shown in the right column); C: CRC tissue (shown in the middle column). Original magnification, ×100-fold. (b) The expressions of ITIH_4_ in CRC tissues compared with the normal colorectal tissues. (c) The IHC scores of the expressions for ITIH_3_ and ITIH_4_; the *p* values were acquired by paired *t*-test. (d, e) The individual line plot diagrams described the IHC scores of ITIH_3_ and ITIH_4_ expressions.

Expression of ITIH_3_ or ITIH_4_ in colorectal cancer and adjacent normal colorectal tissues was analyzed via IHC staining. According to the IHC score assessment, ITIH_3_ expression was dramatically reduced in colorectal cancer, compared with that in normal tissues (*p* < 0.001) (Figures 3(a) and 3(d)). Conversely, ITIH_4_ was upregulated in colorectal carcinoma specimens relative to adjacent normal colorectal tissues (*p* < 0.001) (Figures 3(b) and 3(e)). Analysis of the scores (listed in Figure 3(c)) confirmed that the altered trends in ITIH_3_ and ITIH_4_ expressions between the case and control groups in colorectal tissue are consistent with those in plasma.

## 4. Discussion

While the increasing use of colonoscopy has led to a reduction in mortality of CRC patients [22], more precise and noninvasive methods, such as the identification of reliable blood biomarkers that can stably detect CRC, are essential for improving diagnosis.

CEA is one of the most extensively studied serological tumor markers and has been widely used in the clinical setting, despite the low sensitivity of serum CEA for early-stage CRC [23]. Recently, dozens of more protein biomarkers in serum have been detected for distinguishing the CRC patients from healthy individuals. Among these, TIMP-1, soluble CD26 (sCD26), and M2-pyruvate kinase (M2-PK) have shown relatively promising results [24, 25]. Particularly, TIMP-1 is the only one which has been regarded as an available marker in many clinical researches and could be detected at early stages of CRC. Functionally, TIMP-1 is a multifunctional glycoprotein which can inhibit most matrix metalloproteinases (MMPs) and stimulate tumor growth as well as malignant transformation [26]. Emerging evidence has identified that TIMP-1 is a reliable biomarker with relatively stable and high sensitivity of 65% and specificity of 95%, which exhibits a more superior detecting ability, compared to CEA [26]. As “preclinical development” serum protein biomarkers, the M2-PK and sCD26 still need more evidence for validation [24].

In the present study, we showed for the first time that the plasma concentrations of ITIH_3_ are significantly decreased in CRC patients relative to normal controls (*p* < 0.001), consistent with *ITIH_3_* mRNA expression patterns in tissues of multiple solid cancer types, such as breast, uterus, colon, ovary, lung, and rectum cancers [11]. Earlier, Paris and coworkers revealed the roles of ITIH_1_ and ITIH_3_ in reducing the metastasis of lung cancer in mice while increasing cell attachment *in vitro* [27]. Inhibition of tumor growth and metastasis mediated by ITIH_3_ is related to its stabilizing effects on the extracellular matrix as well as covalent linkage of HA [28]. Therefore, downregulation of ITIH_3_ in plasma appears to be a reasonable step for CRC progression. Data from our experiments support the utility of plasma ITIH_3_ as a potential biomarker for detection of CRC.

The plasma concentration of ITIH_4_ in CRC patients showed a tendency of upregulation (*p* < 0.001). ITIH_4_ protein is closely related to carcinogenesis, development, and metastasis of many solid tumor types. The plasma level of ITIH_4_ is reported to be significantly higher in prediagnostic breast cancer samples and identified as a potential diagnostic marker for breast cancer, consistent with our current findings [29].

In a rat model, ITIH_4_ was upregulated in early intestinal tumors, indicative of a role in extracellular matrix remodeling in colon tumor tissue [16]. In addition, the elevated level of serum ITIH_4_ was associated with early colonic adenomagenesis, which served as the most important premalignant state for CRC [17]. These results indicate that upregulation of plasma ITIH_4_ is closely related to the carcinogenesis of CRC, supporting its utility as an indicator of tumorigenesis in clinical practice.

In our study, no significant differences in ITIH_3_ and ITIH_4_ were observed between the invasive and noninvasive subgroups in CRC patients (*p* values were 0.261 and 0.617, respectively), further supporting the theory that the ITIH_3_/ITIH_4_ biomarker set is related to carcinogenesis rather than prediction of prognosis or metastasis in CRC.

ROC curve analysis was further performed for determining the sensitivity and specificity of plasma ITIH_3_ and ITIH_4_ in distinguishing between CRC patients and healthy subjects. The AUC values of ITIH_3_ and ITIH_4_ were significantly greater than 0.5 (0.638 and 0.801, respectively), supporting their effectiveness in CRC detection. The AUC value of plasma ITIH_4_ was similar to that of the classical biomarker CEA (AUC = 0.816), suggesting that this protein ITIH_4_ can be reliably applied to distinguish CRC. The combination of ITIH_3_ and ITIH_4_ (AUC = 0.827) could be representative of greater effectiveness in disease diagnosis than each protein alone, and the fitting AUC of the two proteins was even higher than that of CEA. In particular, the combination of CEA, TIMP-1, ITIH_3_, and ITIH_4_ would significantly enhance the diagnostic performance (AUC = 0.962), which might provide a more reliable strategy for disease screening.

The NRI analysis showed the range from 6.2% to 17.1% for CRC detective improvement when adding ITIH_3_ and/or ITIH_4_ to TIMP-1, compared with TIMP-1 alone, indicating the highly significant effects of ITIH_3_ and/or ITIH_4_ on diagnosis accuracy improvement.

The statistical differences of circulating ITIH_3_ expression have been detected in the tumors of gastric [30] and pancreatic [31] tumors, compared to healthy controls. Also, the serum expression of ITIH_4_ significantly changed in cancers of hepatocellular carcinoma [32], breast [29], and ovarian [33], in comparison to normal individuals. Therefore, the specificity of ITIH_3_/ITIH_4_ for CRC detection could not reach the absolute value of 100%, due to the changed expression of the two markers across multiple other solid cancers. It should be noted that the quantitative measurements of posttranslational modifications for ITIH_4_/ITIH_3_ might improve the classification of multiple cancers [34], which might improve the specificity of ITIH_3_ and ITIH_4_ in detecting cancers including CRC.

We additionally conducted IHC assessments for the two biomarkers. The IHC score indicated a similar decreasing trend of ITIH_3_ expression along with a dramatic increase in ITIH_4_ expression in CRC tissues relative to that in adjacent normal colorectal tissue. These trends were consistent with serological results, further verifying the reliability of plasma assessments for ITIH_3_ and ITIH_4_.

Kaplan-Meier curves were generated to analyze the prognostic value of ITIH_3_ and ITIH_4_ in CRC metastasis and prognosis (date not shown in results). Notably, the *p* values obtained from the log-rank test for ITIH_3_ and ITIH_4_ (0.570 and 0.511) were not statistically significant, confirming a role of these biomarkers in tumorigenesis rather than prediction of neoplasm metastasis.

The current study has several limitations worth noting. Firstly, a relatively small sample size was examined and further studies with larger sample sizes are thus required for confirmation of our findings. Secondly, only two members of the ITIH family were investigated. Detailed molecular studies should be conducted to clarify the roles and specific mechanisms of ITIH_3_ and ITIH_4_ proteins in tumorigenesis and development of CRC.

In summary, ITIH_3_ is downregulated while ITIH_4_ is upregulated in the plasma of CRC patients, similar to the expression trends observed in CRC tissues. Our findings collectively support the utility of plasma ITIH_3_ and ITIH_4_ proteins as novel tumor biomarkers for diagnosis of CRC.

## Figures and Tables

**Table 1:** The baseline characteristics of patients with colorectal cancer and healthy controls.

Characteristics	CRC patients	Healthy controls	*p* value^∗^
	(*n* = 101)	(*n* = 156)	
Gender			0.363
Female	44	77	
Male	57	79	
Age			0.095
Mean ± SD	61.089 ± 8.505^Δ^	59.359 ± 7.792^Δ^	
Smoking status			0.172
Yes	58	76	
No	43	80	
Drinking status			0.345
Yes	47	82	
No	54	74	
Tumor stage (AJCC)			
Stage I	10		
Stage II	15		
Stage III	19		
Stage IV	57		

^∗^ *p* < 0.05: statistically significant. ^Δ^Years are presented as mean ± SD. CRC: colorectal cancer.

**Table 2:** Serum levels of biomarkers tested in CRC patients in relation to clinicopathological features of tumor.

Variable analyzed	No.	ITIH_3_ (*μ*g/mL)	*p* value^∗^	ITIH_4_ (*μ*g/mL)	*p* value^∗^
		Median (range)		Median (range)	
Age			0.830		0.586
≤60	48	4.429 (2.152–8.170)		0.205 (0.110–0.592)	
>60	53	4.370 (2.852–8.070)		0.213 (0.099–0.415)	
Gender			0.133		0.574
Male	57	4.170 (2.152–8.170)		0.216 (0.106–0.592)	
Female	44	4.577 (2.862–7.098)		0.203 (0.099–0.422)	
Smoking			0.183		0.452
Yes	58	4.204 (2.162–8.170)		0.204 (0.103–0.592)	
No	43	4.569 (2.152–8.070)		0.221 (0.099–0.409)	
Alcohol			0.734		0.240
Yes	47	4.260 (2.152–8.170)		0.222 (0.103–0.592)	
No	54	4.411 (2.352–7.098)		0.207 (0.099–0.421)	
Tumor localization			0.771		0.446
Rectum	34	4.266 (2.152–8.170)		0.206 (0.106–0.429)	
Colon	67	4.385 (2.162–8.070)		0.211 (0.099–0.592)	
Tumor size			0.307		0.872
≤3 cm	45	4.576 (2.352–8.170)		0.210 (0.103–0.592)	
>3 cm	56	4.204 (2.152–8.070)		0.212 (0.099–0.587)	
Tumor stage (AJCC)			0.404		0.278
Stage I	10	4.473 (3.820–4.820)		0.185 (0.110–0.263)	
Stage II	15	4.585 (3.788–8.170)		0.210 (0.127–0.315)	
Stage III	19	4.207 (2.162–6.898)		0.213 (0.162–0.587)	
Stage IV	57	4.219 (2.152–8.070)		0.219 (0.099–0.592)	
Distant metastases			0.261		0.617
Nonmetastatic group	44	4.512 (2.162–8.170)		0.206 (0.110–0.587)	
Metastatic group	57	4.219 (2.152–8.070)		0.219 (0.099–0.592)	
MSI status			0.680		0.692
MSS	55	4.556 (2.162–8.070)		0.211 (0.099–0.592)	
MSI-H	8	4.401 (2.852–5.265)		0.232 (0.127–0.409)	
Unknown	38	4.177 (2.152–8.170)		0.207 (0.103–0.432)	

^∗^ *p* < 0.05: statistically significant. CRC: colorectal cancer; ITIH_3_: inter-alpha-trypsin inhibitor heavy chain H3; ITIH_4_: inter-alpha-trypsin inhibitor heavy chain H4; MSI: microsatellite instability status; MSS: microsatellite stable; MSI-H: microsatellite instability status high.

**Table 3:** The serum concentrations of ITIH_3_ and ITIH_4_ between CRC patients and healthy subjects.

Diagnosis	No. of cases	ITIH_3_ (*μ*g/mL)		ITIH_4_ (*μ*g/mL)	
		Median (range)	*p* value^∗^	Median (range)	*p* value^∗^
CRC patients	101	4.370 (2.152–8.170)	*p* < 0.001^∗^	0.211 (0.099–0.592)	*p* < 0.001^∗^
Healthy controls	156	4.715 (2.665–10.257)		0.134 (0.094–0.460)	

^∗^ *p* < 0.05: statistically significant. CRC: colorectal cancer; ITIH_3_: inter-alpha-trypsin inhibitor heavy chain H3; ITIH_4_: inter-alpha-trypsin inhibitor heavy chain H4.

**Table 4:** The ROC curves for differentiating CRC patients from healthy subjects.

Biomarker	No. of cases (CRC patients/controls)	AUC (95% CI)	Cutoff value	Sensitivity	Specificity	*p* value^∗^
ITIH_3_	101/156	0.638 (0.571-0.704)	4.441 (*μ*g/mL)	0.679	0.525	*p* < 0.001^∗^
ITIH_4_	101/156	0.801 (0.745-0.857)	0.170 (*μ*g/mL)	0.782	0.763	*p* < 0.001^∗^
CEA	101/156	0.816 (0.754-0.878)	3.515 (ng/mL)	0.633	0.897	*p* < 0.001^∗^
TIMP-1	101/156	0.832 (0.776-0.888)	205.680 (*μ*g/mL)	0.723	0.878	*p* < 0.001^∗^
ITIH_3_+ITIH_4_	101/156	0.827 (0.776-0.877)	0.674	0.763	0.851	*p* < 0.001^∗^
ITIH_3_+ITIH_4_+CEA+TIMP-1	101/156	0.962 (0.940-0.985)	0.705	0.917	0.908	*p* < 0.001^∗^

^∗^ *p* < 0.05: statistically significant. CRC: colorectal cancer; ITIH_3_: inter-alpha-trypsin inhibitor heavy chain H3; ITIH_4_: inter-alpha-trypsin inhibitor heavy chain H4; CEA: carcinoembryonic antigen; TIMP-1: tissue inhibitor of metalloproteinase-1.

## Data Availability

The data used to support the findings of this study are available from the corresponding author upon request.
